# Polymorphisms in the Human Inhibitory Signal-regulatory Protein α Do Not Affect Binding to Its Ligand CD47*

**DOI:** 10.1074/jbc.M114.550558

**Published:** 2014-02-18

**Authors:** Deborah Hatherley, Susan M. Lea, Steven Johnson, A. Neil Barclay

**Affiliations:** From the Sir William Dunn School of Pathology, University of Oxford, Oxford OX1 3RE, United Kingdom

**Keywords:** Cancer Therapy, Cell Surface Receptor, Immunology, Leukocyte, Membrane Proteins, Peptide Interactions, Protein Structure, CD47, Inhibitory Receptor, SIRPα

## Abstract

CD47 is a widely distributed membrane protein that interacts with signal-regulatory protein α (SIRPα), an inhibitory receptor on myeloid cells that gives a “don't-eat-me” signal. Manipulation of the interaction is of considerable interest in the immunotherapy of cancer and in xenotransplantation. The amino-terminal ligand binding domain of SIRPα is highly polymorphic in contrast to the single Ig-like domain of CD47. There is confusion as to whether the polymorphisms will affect ligand binding, but this is an important point for this interaction and other paired receptors being considered as targets for therapy. We show by x-ray crystallography that one human SIRPα allele differing in 13 amino acid residues has a very similar binding site and that several different alleles all bind CD47 with similar affinity as expected because the residues are mostly surface-exposed and distant from the binding site. A peptide from the binding site of CD47 has been reported to mimic the CD47 interaction with SIRPα, but we could find no binding. We discuss the possible pitfalls in determining the affinity of weak interactions and also speculate on how SIRPα polymorphisms may have been selected by pathogens and how this may also be true in other paired receptors such as the KIRs.

## Teaser

**Background:** CD47 interacts with SIRPα to down-regulate myeloid cell activity.

**Results:** The extensive polymorphisms in human SIRPα do not affect ligand binding, and a peptide from CD47 does not bind SIRPα.

**Conclusion:** The polymorphisms are being selected for other purposes such as evasion from pathogens.

**Significance:** The polymorphisms are not likely to affect this interaction currently of therapeutic interest.

## Introduction

CD47 is a widely distributed membrane protein that limits the phagocytic activity of macrophages through engagement of the SIRPα^2^ inhibitory receptor as shown by the finding that red blood cells from CD47-deficient mice are rapidly cleared by wild type mice (1–4). The finding that high levels of CD47 was a poor prognostic factor in leukemia (5) has led to many studies suggesting that this interaction may be a valuable therapeutic target (6). One important feature of SIRPα is that its NH_2_-terminal ligand binding domain is highly polymorphic, although its ligand CD47 showed minimal variation (7–9). Analysis of the human SIRPα structure suggested that these polymorphisms would not affect binding of the ligand (10, 11). However, a recent publication suggested that the affinity of these variants for CD47 varied by up to 50-fold (12). This is an important point both because of the interest in this interaction as a therapeutic target and how these polymorphisms came to be selected. The latter has implications for the range of receptors termed “paired receptors” of which SIRPα is a member (1, 13). These are families of membrane proteins expressed mostly on NK and myeloid cells, where one member can give inhibitory signals usually through association of phosphatases via immunoreceptor tyrosine-based inhibition motifs (ITIM) but others give activating signals through the association of adaptors such as DAP12, which recruit kinases through immunoreceptor tyrosine-based activation motifs (ITAM) (13–15). Although some of the polymorphisms on NK paired receptors are known to reflect specificity for binding to MHC antigens, others could reflect pathogen pressure.

We report the x-ray crystallographic structure of a complex of CD47 with a common allele of SIRPα that differs in 13 residues in the NH_2_-terminal domain to that already determined. We found that the affinities of the variants of SIRPα for CD47 are very similar. We also tested a peptide mimic from CD47 being evaluated as a possible therapeutic (12), but we found no binding. The results are discussed together with an analysis of the variability in other paired receptors and implications for therapeutics and evolution.

## EXPERIMENTAL PROCEDURES

### 

#### 

##### Crystallization of SIRPα-CD47 Complex, Data Collection, Structure Determination, and Refinement

Recombinant CD47 Ig superfamily domain and the NH_2_-terminal domain of SIRPαv1 (residues 1–149; accession number NP_542970) were produced in CHO Lec3.2.8.1 cells as described previously (11). The proteins were purified by nickel affinity chromatography and gel-filtrated in 10 mm HEPES, pH 7, 150 mm NaCl, 0.02% NaN_3_. CD47 and SIRPαv1 were mixed in a 1:1 molar ratio, deglycosylated using endoglycosidase Hf, and concentrated to contain each protein at ∼620 μm. Sitting drop vapor diffusion crystallization experiments were performed using an OryxNano robot to dispense nanoscale protein precipitant drops that were equilibrated against precipitant reservoirs at 12 °C. Crystals of the CD47-SIRPαv1 complex grew from 300-nl drops containing 50% protein from 0.1 m Tris, pH 8.5, 20% w/v PEG 6000. Crystals were cryoprotected in mother liquor supplemented with 15% glycerol and flash-frozen in liquid nitrogen. Diffraction data were collected at the European Synchrotron Radiation Facility (ESRF, Grenoble, France) at a wavelength of 0.97930 Å and were processed using Xia2 (16). The structure was determined by molecular replacement using Phaser (17) with the CD47-SIRPαv2 structure (PDB code 2jjs) as a search model. Buccaneer (18) was used to autobuild followed by iterative cycles of refinement with autoBuster and manual model building in COOT (19, 20).

##### Affinity Measurements of SIRPα Variants and CD47

The variants in human SIRPα were introduced by PCR into the pEFBOS vector (21) containing the three Ig superfamily domains of SIRPα linked to rat CD4d3 and -4 as an antigenic label and a sequence to allow biotinylation (22). The proteins were expressed by transient transfection in 293T cells, biotinylated, and immobilized on a BIAcore chip to which streptavidin had been coupled in a BIAcore^TM^ 3000 at 37 °C (23). Dilutions of recombinant CD47 extracellular domain (see above) were passed over the variants, and the affinity was determined from equilibrium binding as described previously (24). SIRPα binding to CD47 peptides was tested in a similar manner by immobilizing biotin aminohexanoic acid-GNYTCEVTELTREGETIIELK (linear peptide) and biotin aminohexanoic acid-CEVTELTREGEC (cyclized peptide) (synthesized by Peptide Protein Research Ltd., United Kingdom) and passing over dilutions of recombinant SIRPα. Rat CD4 d3 and 4-biotin or biotin-EFLTIpYEDVKD were immobilized as control protein or peptide, respectively.

##### NH_2_-terminal Protein Sequencing

The NH_2_ sequence of recombinant SIRPαv2 and SIRPαv10 was determined by protein sequencing by AltaBioscience, Birmingham, UK.

## RESULTS

### 

#### 

##### Structure of the NH_2_-terminal Domain of SIRPα(v1) in Complex with the Ig-like Domain of CD47

**FIGURE 1.**
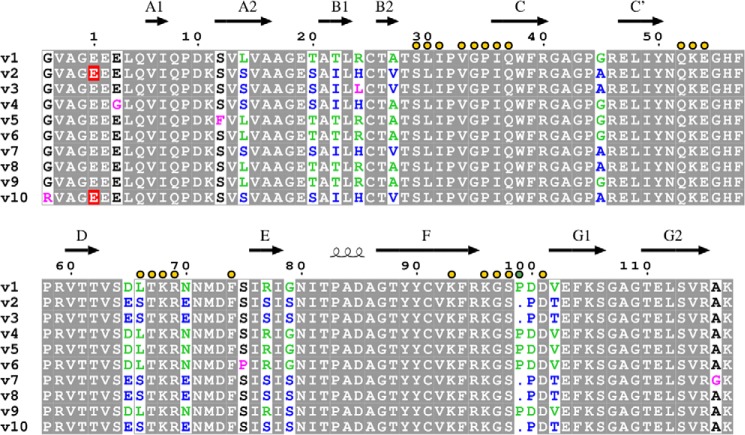
**Sequence alignment of the variants of SIRPα based on structural alignment of v1 and v2.** The sequences are from Ref. 7. Variants v1 and v2 are the most common (accession numbers NP_542970 and CAA71403). The residues that differ between these variants are marked in *green* (v1) and *blue* (v2). The majority of variants contain residues found in either v1 or v2 with the exceptions shown in *pink*. The NH_2_ terminus is designated residue 1 and has been determined for v2 and v10 (shown in *red*). The positions of the β-strands are indicated as determined for v1 (see below and v2 (11)). Residues making direct and indirect contacts with CD47 are denoted by *yellow dots* above the sequence. The *green dot* represents the water-mediated hydrogen bond formed by Pro-99 with Lys-39 and Asp-46 of CD47 seen only in the CD47-SIRPα v2 complex.

**FIGURE 2.**
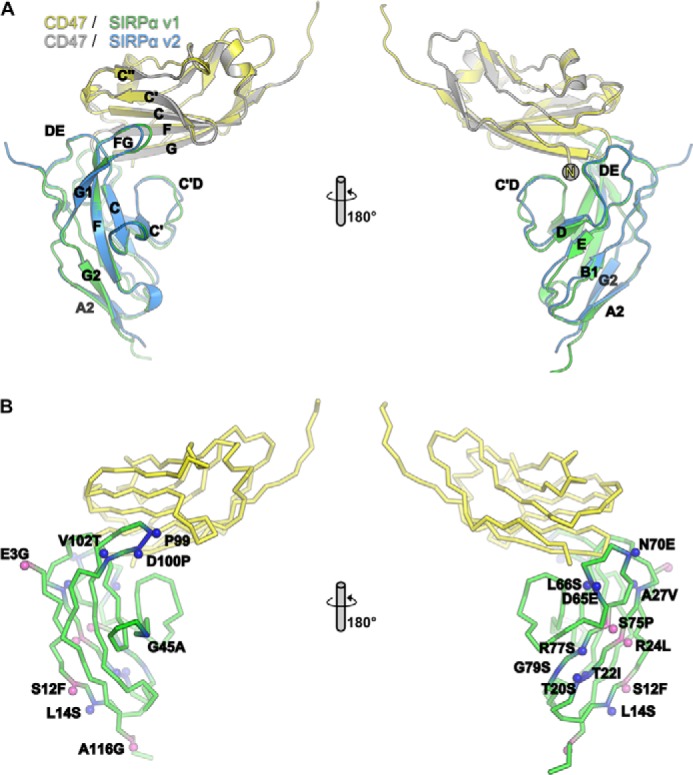
**Interaction of SIRPα with CD47.** *A,* structure of domain 1 of SIRPαv1 (*green*) in complex with the Ig- like domain of CD47 (*yellow*) determined by x-ray crystallography to a resolution of 1.92 Å. The CD47-SIRPαv1 complex is superposed on the CD47 (*gray*)/SIRPαv2 (*blue*) structure (PDB code 2jjs, chains A and C). Two views are shown rotated 180° on the *y* axis. *B,* mapping of the positions where SIRPα variants are polymorphic onto the CD47-SIRPαv1 structure using the same color coding as in Fig. 1.

The two most common alleles of SIRPα (v1 and v2) are also the most divergent in sequence with 13 residues differing in the NH_2_-terminal ligand binding domain (total length 120 residues) (Fig. 1). To determine the structural consequences of the sequence differences, the structure of the NH_2_-terminal domain of SIRPαv1 was determined in complex with its ligand CD47 to a resolution of 1.92 Å (Table 1). The overall structure (Fig. 2) was very similar to that previously described for CD47-SIRPαv2 (PDB; 2jjs) (11) with a root mean square deviation of 0.65 Å over 231 Cα atoms. The interaction interfaces of SIRPαv1 and SIRPαv2 with CD47 are almost identical with only minor differences in the DE and FG loops. In the DE loop of SIRPα, v1 has a Leu at position 66, whereas v2 has Ser, both of which interact with the NH_2_-terminal pyroglutamic acid of CD47. Ser-66 (v2) forms main chain and side chain hydrogen bonds with the pyroglutamic acid lactam ring, whereas in v1 only the main chain hydrogen bond is possible. Although the FG loop of v1 is longer than v2 due to the insertion of an Asp (Asp-100), v1 mediates the same interactions with CD47 as seen in the structures for v2 in complex with CD47. However, the SIRPαv1 main chain oxygen atom of Pro-99 can form water-mediated hydrogen bonds with Asp-46 and Lys-39 of CD47.

**TABLE 1:** **Data collection and refinement statistics of SIRPα(v1)-CD47 complex**

**Data collection statistics**	
Beamline	ID23-1 (ESRF)
Wavelength	0.97930 Å
Resolution limits	34.62 to 1.92 Å
Space group	P2
Unit cell dimensions±	67.39 Å, 32.47 Å, 69.61 Å; 90°, 115°, 90°
Total no. of observations	75,123
Unique reflections	21,067
Multiplicity	3.6 (3.0)
Completeness	99.1% (96.0%)
*I*/σ(*I*)	12.9 (2.5)
*R*_merge_*^a,b^*	5.3% (44.4%)
Processing programs	XIA2

**Refinement statistics**	
Resolution limits	34.62 to 1.92 Å
No. of reflections in working set	21,066
No. of reflections in test set	1081
*R*_work_*^a,c^*	0.1926 (0.2309)
*R*_free_*^a,d^*	0.2279 (0.2539)
No. of atoms (protein/carbohydrate/water)	1855/56/174
Residues in Ramachandran favored region	98.7%
Ramachandran outliers	0.0%
r.m.s.d.*^e^* bond lengths	0.011 Å
r.m.s.d. bond angles	1.21°
Average *B* factors (protein/carbohydrate/water)	39/67/44 Å^2^

*^a^* Numbers in parentheses refer to the appropriate outer shell.

*^b^ R*_merge_ = 100 × (Σ*_hkl_* Σ*_i_*|*I*(*hkl*) − 〈*I*(*hkl*)〉|/Σ*_hkl_* Σ*_i_I_i_*(*hkl*)), where *I*(*hkl*) is the intensity of an individual measurement of a reflection, and 〈*I*(*hkl*)〉 is the average intensity of that reflection.

*^c^ R*_work_ = (Σ*_hkl_*‖*F*_obs_| − |*F*_calc_‖/Σ*_hkl_*|*F*_obs_|), where |*F*_obs_| and |*F*_calc_| are the observed and calculated structure factor amplitudes.

*^d^ R*_free_ equals the *R*-factor of test set (5% of the data removed prior to refinement).

*^e^* r.m.s.d. is root mean square deviation from ideal geometry.

##### Affinity of SIRPα Variants for CD47

**FIGURE 3.**
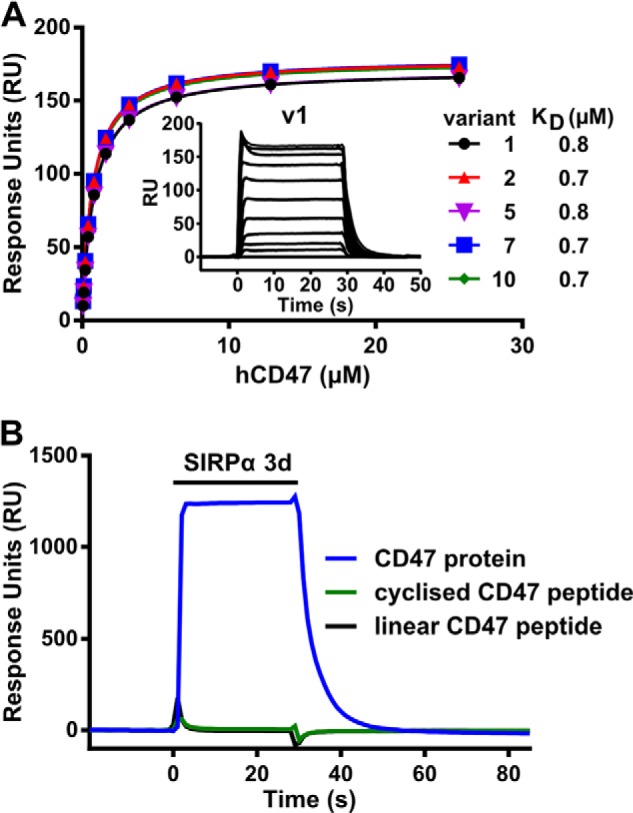
**Determination of affinities of SIRPα variants by surface plasmon resonance.** *A,* recombinant SIRPα variants were immobilized via a biotin tag to a streptavidin-coated CM5 BIAcore chip. Varying concentrations of soluble monomeric CD47 were injected over the proteins. The specific binding responses were plotted, and the equilibrium affinity constants (*K_D_*) were calculated from nonlinear curve fitting. A representative experiment is shown. *Inset* is a representative graph of the equilibrium binding responses of SIRPα v1 with 0.05 to 25.7 μm CD47. *B,* reverse experiment showing SIRPα (38 μm) binding to immobilized CD47 but neither of the two CD47 peptides.

The affinities of SIRPαv1 and SIRPαv2 for CD47 were determined by surface plasmon resonance together with three other variants that were reported to have significantly different affinities (varying by 50-fold) (Fig. 3*A* and Table 2)(12). The variants differ mostly in surface residues on the common Ig-like domain and distal from the binding site (Fig. 2), so from a protein structural point of view, the suggestion that these have different affinities is unexpected. However, the values we determined for the affinities of the other SIRPα variants for CD47 are not significantly different from that determined for v1 (Tukey's multiple comparisons test) and are close to previously determined values (Table 2) (11, 25, 26). The most likely explanation for the findings of Rodriguez *et al.* (12) is in their use of an indirect assay giving nonquantitative results due to factors other than direct binding activity, as the assay involved binding of recombinant biotinylated CD47 and then detection with antibodies against biotin. Thus, the binding observed in Ref. 12 is probably due to the presence of some aggregated CD47 protein leading to avidity effects with the bivalent antibody. In addition, the v10 is identical to v2 apart from an Arg that is predicted to be in the signal sequence (residue −4) (Fig. 1) (7). The NH_2_-terminal protein sequencing confirmed the sequence was EEELQV for both v2 and v10.

**TABLE 2:** **Affinity measurements for CD47 binding SIRPα variants** Affinity measurements for CD47 binding SIRPα variants are shown. ND means not detectable. The values for the affinities determined by BIAcore are the mean ± S.D. from at least three independent experiments at 37 °C. SIRPαv2, CAA71403, and SIRPαv1, NP_542970.

SIRPα variant no.*^a^*	Affinity (*K_D_* μm) from Ref. 12	Affinity (*K_D_* μm) determined by BIAcore (this study)
(v1) SIRPα	0.46	0.74 ± 0.07
(v2) SIRPα	0.44	0.64 ± 0.06
(v5)	2.50	0.78 ± 0.09
(v7)	3.21	0.65 ± 0.05
(v10)	0.07	0.67 ± 0.05
SIRPα for CD47 peptide	0.16	ND

*^a^* Nomenclature of variants is according to Ref. 7 and is also used in Ref. 12.

##### CD47 Peptide Binding to SIRPα

There is considerable interest in using CD47 as a “don't-eat-me” signal to improve the stability of particles delivering drugs by inhibiting clearance by phagocytosis; either native CD47 is used (27) or more controversially a peptide mimic of the binding site of CD47 (12). Although linear peptides can be substrates for recognition by protein domains in, for example, the recognition of phosphotyrosine motifs by SH2 domains and the recognition of RGD motifs by integrins, the mimicking of highly convoluted protein-protein interactions, such as those between CD47 and SIRPα, would be highly significant in terms of targeting this interaction and indeed the large number of other interactions between cell surface proteins. We tested the binding of SIRPα to two of the CD47 peptides used in Ref. 12 by immobilizing them in the same way through the NH_2_-terminal biotin to a BIAcore chip and passed over recombinant CD47. In contrast to their finding that the peptide had comparable affinity to the whole protein, we found no significant binding (Fig. 3*B*).

## DISCUSSION

**FIGURE 4.**
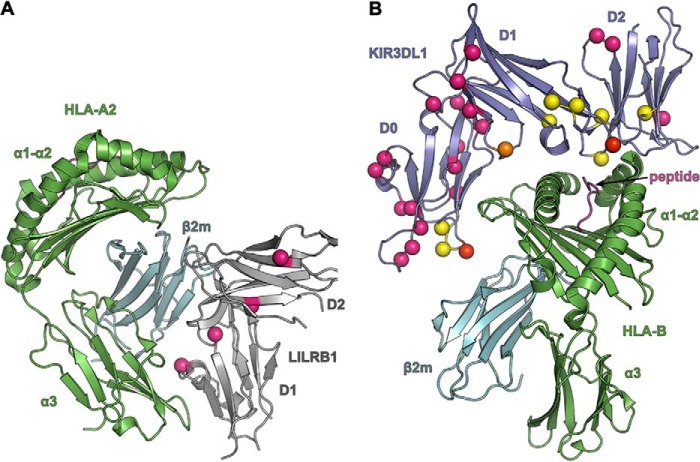
**Polymorphic residues in paired receptors.** *A,* positions of four polymorphic residues in LILRB1 are indicated in *pink*. LILRB1 is colored *gray*; the MHC antigen (HLA-A2) is in *green,* and β2-microglobulin (β*2m*) is colored *cyan.* Data are from Ref. 30 and PDB code 1p7q. *B,* positions of polymorphic residues in KIR3DL1 (colored *purple*) unlikely to affect binding are colored *pink*; those that might affect binding are *yellow*; those forming direct contacts are *red,* and a water-mediated contact is in *orange*. Data are from Ref. 33 and PDB code 3vh8.

The polymorphisms in human SIRPα lead to changes in surface-exposed amino acids, but we show this does not affect binding to CD47, which shows no comparable polymorphisms. We suggested that these polymorphisms may be the result of selection following binding of pathogens or pathogen products that target the inhibitory receptor as it would be advantageous for pathogens to down-regulate the activity of myeloid cells (10). This may be a general mechanism for other paired receptors (13, 15), and recent data on NK receptors supports this. Both LILRB1 and inhibitory KIRs recognize polymorphisms in MHC (28, 29) and in addition have many polymorphisms distal to the binding site, which in some cases are known not to affect binding (Fig. 4) (30). Some of these polymorphisms may be important in establishing levels of expression (31), but it seems plausible that some are the result of selection by resistance to pathogens. Recent analysis of the KIR has shown that two families can be distinguished as follows: the one containing more activating genes is more common where infections are common and the other is more common in developed countries where it is proposed that this correlates with improved human reproduction (32). This supports the idea that pathogens target the inhibitory member of paired receptors following gene duplication and mutation and that variants in the inhibitory receptor are selected together with activating genes that might act as a counterbalance to the successful targeting of the inhibitory receptor (10).

## Footnotes

2 The abbreviations used are:

SIRP: signal-regulatory protein
PDB: Protein Data Bank.
